# pH Changes Have a Profound Effect on Gene Expression, Hydrolytic Enzyme Production, and Dimorphism in *Saccharomycopsis fibuligera*

**DOI:** 10.3389/fmicb.2021.672661

**Published:** 2021-06-24

**Authors:** Mohamed El-Agamy Farh, Najib Abdellaoui, Jeong-Ah Seo

**Affiliations:** School of Systems Biomedical Science, Soongsil University, Seoul, South Korea

**Keywords:** *Saccharomycopsis fibuligera*, *nuruk*, transcriptome analysis, yeast cell-hyphae transition, pH effect, starch and cellulose hydrolysis

## Abstract

*Saccharomycopsis fibuligera* is an amylolytic yeast that plays an important role within *nuruk* (a traditional Korean fermentation starter) used for the production of *makgeolli* (Korean rice wine), which is characterized by high acidity. However, the effect of pH change (neutral to acidic) on the yeast cell to hyphal transition and carbohydrate-hydrolyzing enzyme activities for *S. fibuligera* has not been investigated yet. In this study, *S. fibuligera* strains were cultured under the different pH conditions, and the effect on the enzyme production and gene expression were investigated. An acidic pH induced a hyphal transition from yeast cell of *S. fibuligera* KPH12 and the hybrid strain KJJ81. In addition, both strains showed a gradual decrease in the ability to degrade starch and cellulose as the pH went down. Furthermore, a transcriptome analysis demonstrated that the pH decline caused global expression changes in genes, which were classified into five clusters. Among the differentially expressed genes (DEGs) under acidic pH, the downregulated genes were involved in protein synthesis, carbon metabolism, and *RIM101* and cAMP-PKA signaling transduction pathways for the yeast-hyphal transition. A decrease in pH induced a dimorphic lifestyle switch from yeast cell formation to hyphal growth in *S. fibuligera* and caused a decrease in carbohydrate hydrolyzing enzyme production, as well as marked changes in the expression of genes related to enzyme production and pH adaptation. This study will help to elucidate the mechanism of adaptation of *S. fibuligera* to acidification that occur during the fermentation process of *makgeolli* using *nuruk*.

## Introduction

The Korean traditional wine *makgeolli* is made by fermenting the starch materials of rice (Jang, 1989; Yeo and Jeong, 2010). High-quality *makgeolli* is characterized by high acidity and alcohol content (Song et al., 2015). Over 100 traditional *makgeolli* brands are available, and they are worth millions of dollars on the market (Jang, 1989; Yeo and Jeong, 2010). To produce *makgeolli*, the fermentation starter *nuruk* needs to be prepared from several kinds of grains, especially rice grains, using airborne microorganisms. These microorganisms provide a source of several hydrolytic enzymes that are required for starch degradation during saccharification to produce glucose and other organic acids (Song, 2013; Yang et al., 2013; Bal et al., 2014, 2015; Kim et al., 2014; Park et al., 2016). Then, glucose is fermented by *Saccharomyces cerevisiae* to produce alcohol. Thus, the quality of alcohol in *makgeolli* critically depends on the characteristics of the *nuruk* used for fermentation. The saccharification capability is an essential index used to estimate the quality of the *nuruk* (Lee et al., 2017), and it is based on microorganisms that occupy the *nucleus* and produce hydrolytic enzymes (Lee et al., 2017). Currently, several *nuruk*-inhabiting fungi, yeasts, and bacteria have been isolated from traditional *nuruk* and used to make commercial *nuruk* products (Jo and Lee, 1997; Seo et al., 2007; Carroll et al., 2017).

Since *nuruk* ingredients are mainly composed of polysaccharides, predominant microorganisms capable of degrading polysaccharides in *nuruk* include filamentous fungi, such as *Rhizopus oryzae* and *Aspergillus oryzae* (Kim et al., 2014). Because of the temperature increase during fermentation, the heat-resistant strain *A. oryzae* has an advantage over the heat-labile strain *R. oryzae* (Yang et al., 2011). *A. oryzae* secretes amylases at high levels (Norihiro et al., 1989; Liu et al., 2014) and is thus an important fungus for the saccharification step (Yang et al., 2011, 2013; Song, 2013). There have also been attempts to use alternative and more beneficial and risk-free yeasts, such as *Monascus* (Shin et al., 2017) and *Saccharomycopsis fibuligera* (Park et al., 2014). Of the microorganisms involved in *nuruk* fermentation, another important microorganism is *S. fibuligera*, a member of Saccharomycotina, which is a subphylum of Ascomycota. *S. fibuligera* is characterized by a morphology-switching lifestyle known as dimorphism and can propagate by branched hyphal growth or yeast cell-like growth (Kurtzman and Smith, 2011). *S. fibuligera* is considered the best producer of amylolytic enzymes among all ascomycetous yeasts (de Mot et al., 1984). It was found to be a predominant yeast in various Asian fermentation starters, including *nuruk* in Korea (Bal et al., 2014; Carroll et al., 2017; Farh et al., 2017). It produces cellulose-degrading enzymes that play a key role in the saccharification process of lignocellulosic compounds (Ma et al., 2015; Van Zyl et al., 2016). Ethanol can be produced from carbohydrate-type substrates by cocultivation of *S. fibuligera* with ethanol producers such as *S. cerevisiae* and *Zymomonas mobiliz* (Abouzied and Reddy, 1987; Reddy and Basappa, 1996; Park et al., 2014).

Saccharification by *nuruk* mostly produces simple sugars. Glucose is mainly fermented by *S. cerevisiae*, while other sugars can be converted by other microorganisms into organic acids, resulting in a decline in pH from neutral to acidic. The pH is weakly basic to neutral at the starting point, suddenly becomes strongly acidic (pH 3.31–2.96), and slightly increases to 3.34 at the end of the fermentation process (Song et al., 2015). The dynamic pH change during fermentation could affect the growth, morphology and physiology of microorganisms involved in fermentation. Some microorganisms, such as *Sclerotinia sclerotiorum*, a plant necrotrophic pathogen, and *Ustilago maydis*, a filamentous and pathogenic form of the dimorphic biotrophic pathogen, grow well under acidic pH (Cotton et al., 2003), whereas other microorganisms, such as *Candida albicans* and *Cryptococcus neoformans*, show better growth under alkaline pH conditions (Davis, 2003; O’Meara and Alspaugh, 2012). In addition, certain environmental microorganisms, such as *Yarrowia lipolytica*, favor dimorphic growth under ambient pH conditions but secrete protein-degrading enzymes under both acidic and alkaline pH conditions (Gonzalez-Lopez et al., 2002; Ruiz-Herrera and Sentandreu, 2002). Thus, environmental pH greatly influences the growth and activity of microorganisms. Microorganisms should sense the environmental pH and adapt to the conditions. To understand such adaptation, signaling cascades have been elucidated in various types of yeast and filamentous fungi at the molecular level, and they consist of MAPK, cAMP-PKA, and PacC/Rim101. MAPK and cAMP-PKA have also been shown to regulate dimorphic growth and morphology-dependent pathogenesis (Leberer et al., 2001; Martínez-Espinoza et al., 2004; Kozubowski et al., 2009), whereas PacC/Rim101 is involved in the regulation of adhesins, secretion of hydrolytic enzymes, and resistance to sodium and lithium (Cotton et al., 2003; Cornet and Gaillardin, 2014).

The pH change occurs during fermentation in the process of rice wine production. However, little is known about the effect of pH change on the growth and activity of microorganisms that grow in *nuruk*, such as *S. fibuligera*. In this study, we examined the effect of dynamic pH changes on the growth and physiology of *S. fibuligera* strain KPH12 and its newly discovered interspecies hybrid KJJ81 (Choo et al., 2016) by focusing on hydrolytic enzyme production, dimorphism, and gene expression. We provided evidence that KPH12 and the hybrid strain KJJ81 of *S. fibuligera* showed a morphological switch from the yeast-like form to hyphae-like elongated form in response to pH reduction. In addition, we identified the gene classes that were dramatically upregulated and downregulated due to the pH change occurring during fermentation.

## Materials and Methods

### Strains, Medium, and Growth Conditions

*Saccharomycopsis fibuligera* KJJ81 and KPH12 strains were obtained from the original authors and used in this study. Here, the strains were routinely cultured on yeast extract peptone glucose (YPG) agar medium [2% (w/v) glucose, 1% (w/v) yeast extract, 1% (w/v) peptone, 2% (w/v) agar, pH 6.0] which was modified with YEPD described as the previous report (Zhu et al., 2017). To test the pH effect on hydrolytic enzyme secretion, yeast cells of each strain (10^7^ CFU/ml) were spotted on YPG agar buffered with phosphate buffer (0.2 M disodium hydrogen phosphate, and 0.2 M sodium dihydrogen phosphate) for pH 7.0 and citrate buffer (0.1 M citric acid, and 0.1 M sodium citrate) for pH 6.0, 5.0, 4.0, and 3.0. Three sets of buffered medium were inoculated with each strain; the first set was supplemented with 0.2% (w/v) starch to assess starch degradation, the second set was supplemented with 0.2% (w/v) carboxymethylcellulose (CMC) to estimate cellulose degradation, and the third set was supplemented with 2% (w/v) skim milk to estimate protein degradation. The inoculated plates were incubated at 25°C. Starch degradation was examined 3 days after incubation, and cellulose and protein degradation was examined 5 days after incubation. To test the pH effect on dimorphic growth, yeast cells of both strains (10^6^ CFU/ml) were cultured in YPG broth buffered as described above. The inoculated broths were incubated at 25°C with shaking (200 rpm), and the cell morphology was examined 20 h after incubation; the proportion of yeast and hyphal cells was measured under a light microscope as described for *Y. lipolytica* (Ruiz-Herrera and Sentandreu, 2002). To test whether the pH or glucose concentration plays a role in hydrolytic enzyme production, particularly those for starch and cellulose degradation, the yeast cells of both strains were spotted on YPG agar [2% (w/v)] plates supplemented with 0.1, 0.5, 2, or 10% (w/v) glucose. For each concentration, one type of substrate was added to estimate the degradation under three different buffered conditions: non-buffered, pH 7-buffered (using phosphate buffer), and pH 3-buffered (using citrate buffer) conditions. To test the effect of glucose or pH on dimorphism regulation, yeast cells of both strains were grown on YPG broth supplemented with the glucose concentrations set mentioned above under the same three buffered conditions. To confirm the involvement of cAMP-PKA in hyphal growth induction, the yeast cells of both strains were grown in pH 4-buffered YPG broth and agar supplemented with 600 μM clozapine, a cAMP-PKA blocker. Clozapine was filter-sterilized and added to autoclaved medium after cooling. Cells were cultured on clozapine-free broths and plates as controls. The inoculated plates were incubated at 25°C for 5 days, while inoculated broths were incubated at 25°C with shaking (200 rpm) for 20 h. Hyphae development was examined using a light microscope.

### Scanning Electron Microscopy (SEM)

Cells grown in YPG at different pH values starting from pH 7.0 to pH 3.0 were collected by filtration and prepared for SEM examination as described previously (Staniszewska et al., 2013) with some modifications. Cells were suspended in fixation buffer [2.5% (w/v) glutaraldehyde in 0.2 M cacodylate buffer, pH 7.2–7.4] for 19 h at 4°C. Samples were washed three times with 0.05 M sodium cacodylate buffer and subsequently suspended in 2% (w/v) osmium tetroxide and 0.1 M cacodylate buffer (1:1, v/v) for further fixation. Afterward, initial dehydration of cells was carried out by placing them in serial concentrations of 50 and 70% ethanol two times for 10 min each; 95% ethanol two times for 5 min each; and 100% ethanol two times for 1 min each. The final dehydration step was carried out by placing the samples in liquid CO_2_ for 90 min following the critical drying point method. Subsequently, the samples were subjected to silver coating under vacuum evaporation and examined by SEM.

### RNA-Seq Analysis

To examine the whole transcriptional responses at each pH point, yeast cells were grown in YPG broth buffered at different pH values starting from 7.0 to 3.0 as described above. Cells were collected 18 h after incubation using vacuum filtration and rapidly stored at –80°C. Total RNA was prepared using an easy-spin total RNA extraction kit (iNtRON, South Korea), and sequencing of the total RNA was performed using an Illumina HiSeq 2500 instrument (Illumina, United States). Complementary DNA (cDNA) libraries were constructed at Theragen Etex Bioinstitute (Seoul, South Korea). Pair-end RNA sequencing was performed by the company as reported previously (Mardis, 2008). Clean reads with high nucleotide accuracy were aligned with the genomes of either KPH12 or KJJ81 using Burrows-Wheeler Aligner (BWA) (Li and Durbin, 2009), and the total counting, expressed as RPKM (reads per kilobase of transcript, per million mapped reads), of the aligned fragments with the exon parts of the genes was determined using HTSeq (Anders et al., 2015). Counts were normalized to be expressed as RPKHM (reads per kilobase of transcript per hundred million mapped reads). The predicted protein was blasted against the NCBI database of non-redundant protein sequences (nr), Uniprot, and relative fungi RefSeq (i.e., *S. cerevisiae*, *C. glabrata*, *A. oryzae*, and *S. pombe*) databases with a E-value cutoff of 10^–10^. The raw data were filtered to remove the genes with zero reads among all samples and then transformed. Filtration and transformation were performed using the R package EdgeR (Robinson et al., 2010). A multidimensional scaling (MDS) plot was generated to show the variation in the expression of genes in the samples using the R package *mixOmics* (Rohart et al., 2017). Differentially expressed genes (DEGs) were determined by EdgeR, and *P*-values were controlled using the false discovery rate (FDR) (Benjamini and Hochberg, 1995) with a threshold lower than 0.05.

For the clustering analysis, the average RPKM of the DEG lists of KPH12 and KJJ81 was clustered using the R package *Mfuzz*. Five clusters were identified, and the fuzzifier coefficient was set to optimal. Following the clustering analysis, genes in each cluster with memberships lower than 0.5 were excluded and the remaining genes were subjected to a Gene Ontology (GO) enrichment analysis. GO enrichments of chosen clusters were filtered using the online public website REVIGO, and the resulting GO terms were visualized on Cytoscape.

### Reverse Transcription, PCR, and Quantitative Reverse Transcription-PCR (qRT-PCR)

Prior to sending the RNA samples for RNA sequencing, aliquots were obtained for qRT-PCR validation of the gene of interest. Samples were kept at –80°C until cDNA synthesis. Only samples at pH 7 and pH 3 were considered for the qRT-PCR validation for the gene of interest. cDNAs were synthesized using SuperScript III First-Strand (Invitrogen, United States) according to protocols provided by the manufacturer. Two micrograms of total RNA was mixed with the synthesis mixture at a volume of 20 μl, and the total volume was further diluted to 50 μl for qRT-PCR. Primer pairs of target genes were designed manually after aligning the gene sequences of the KPH12 and A and B genomes of KJJ81 using ClustalX2 (Larkin et al., 2007) and chemically synthesized (Macrogen Inc., Seoul, South Korea). The primers used in this study are listed in Supplementary Table 7. Quantitative real-time PCR (qRT-PCR) was carried out in a 10 μl reaction volume consisting of 1 μl cDNA, 10 pmol each of forward and reverse primers, 5 μl 2X iQ^TM^ SYBR^®^ Green Supermix (BioRad, United States), and water to the final volume. Reactions were carried out using a CFX Connect^TM^ Real-Time System (Bio-Rad, United States) in a Hard-Shell^®^ 96-Well PCR Plate (Bio-Rad, United States). Among the well-known housekeeping genes, *Y*-tubulin (*TUB4*) was selected as a control since it showed the most stable expression throughout all pH values. The following thermal cycler conditions were based on the manufacturer’s recommendations: 3 min at 95°C; 39 cycles at 95°C for 10 s and the proper annealing temperature for 30 s; and one more cycle starting from 65 to 95°C for 5 s in 0.5°C increments for the melt curve analysis. The fluorescent product was detected during the annealing step of each cycle. Amplification, detection, and data analysis were performed on a CFX Manager^TM^ Maestro version 1.0 (Bio-Rad, United States). The relative fold differences in template abundance for each sample were determined by deducting the C_*T*_ value of each gene expression from the C_*T*_ value of *TUB4*.

### Statistical Analysis

All data were analyzed using SPSS v. 26.0 software (SPSS Inc., Chicago, IL, United States). The statistical analysis includes comparison of group mean values with one-way ANOVA, and multiple comparisons among the groups with Scheffe *post-hoc* test at a significance level of *p* < 0.05.

### Accession Numbers

The RNA-seq raw data were deposited in the BioSample database of NCBI. The accession numbers are serial numbers from SRX10182890 to SRX10182919.

## Results

### Acidic pH but Not Glucose Causes Dimorphic Growth of *S. fibuligera*

**FIGURE 1:**
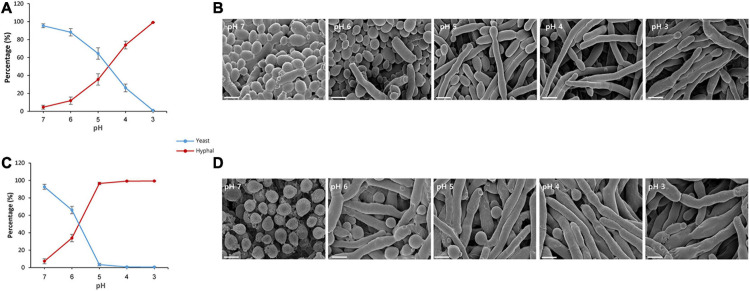
Yeast-to-hyphae transition of *Saccharomycopsis fibuligera* at acidic pH values. KPH12 strain **(A,B)** and KJJ81 interspecies hybrid strain **(C,D)** were grown at the indicated pH values. **(A,C)** Percentage of yeast and mycelium in *S. fibuligera* grown in YPG broth buffered at pH 7, 6, 5, 4, 3 and incubated at 25°C for 20 h with shaking at 200 rpm. *Blue*, yeast percentage; *Red*, hyphae percentage. **(B,D)** Morphology and size of the cells at each pH value; scale bar = 4 μm.

The effect of the pH change occurring during fermentation on the growth of *S. fibuligera* was investigated by growing *S. fibuligera* on YPG medium with different pH values, and we examined the morphology. Various pH values were used to mimic the pH change occurring during fermentation. Both the KPH12 and KJJ81 strains of *S. fibuligera* were grown on these plates and observed for morphological changes. For both the KPH12 and KJJ81 strains, when the pH of the medium was decreased from pH 7 to pH 3, the number of yeast-type cells gradually decreased, and concomitantly, the number of cells with hyphae increased (Figures 1A,C), indicating that a decline in the pH value induced the dimorphic growth of *S. fibuligera*. The single cells started to elongate at pH 6 for both strains, indicating that hyphal growth is induced at pH 6. The interspecies hybrid strain KJJ81 was more responsive to the change in pH in terms of hyphal growth than KPH12 (Figures 1B,D).

**FIGURE 2:**
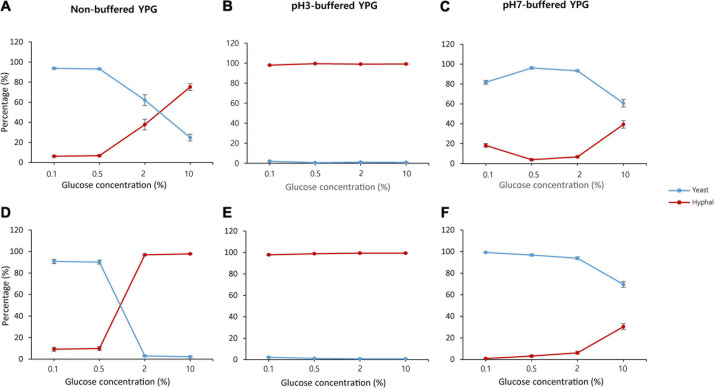
Percentage of yeast and mycelium in *Saccharomycopsis fibuligera*. KPH12 strain **(A–C)** and KJJ81 interspecies hybrid strain **(D–F)** were grown in YPG broth supplemented with 0.1, 0.5, 2, and 10% glucose in non-buffered conditions **(A,D)**, pH 3-buffered conditions **(B,E)**, and pH 7-buffered conditions **(C,F)**. *Blue*, yeast percentage; and *red*, hyphae percentage.

Previously, we showed that the glucose concentration controls the dimorphic growth and hydrolytic enzyme production of *S. fibuligera* KPH12 and KJJ81 (Choo et al., 2016). During fermentation, the glucose concentration also changes because of starch or carbohydrate degradation in *nuruk*. Thus, to obtain a better understanding of the underlying cause of the dimorphic switch in the growth of *S. fibuligera* during fermentation, we examined the effect of glucose concentration on dimorphic changes. Under non-buffered conditions, both strains showed that the yeast-to-hyphae switch was induced when the glucose concentration changed from 0.1 to 10% (Figure 2 and Supplementary Figure 1). Yeast cells were dominant at the lowest glucose concentration, whereas hyphal cells were dominant at the highest glucose concentration (Figures 2A,D and Supplementary Figures 1A,C). Since *S. fibuligera* was grown under non-buffered conditions, the pH may have changed during the incubation and subsequently affected the yeast-to-hyphae switch. Thus, to assess the pH effect on the yeast-to-hyphae switch, we measured the pH at each glucose concentration 20 h after incubation with both strains and found that the pH was changed from neutral to weakly basic in 0.1% glucose, whereas the pH was acidic in 2–10% glucose (Supplementary Figures 1B,D). Both strains developed complete hyphal growth under buffered conditions at pH 3 (Figures 2B,E and Supplementary Figures 1A,C) and yeast growth (Figures 2C,F and Supplementary Figures 1A,C) regardless of the glucose concentration. These results suggest that both carbohydrate degradation and dimorphism are regulated by pH change and not by glucose concentration.

### pH Change (Not Glucose Concentration) Has a Suppressive Effect on the Secretion of Hydrolytic Enzymes From *S. fibuligera*

**FIGURE 3:**
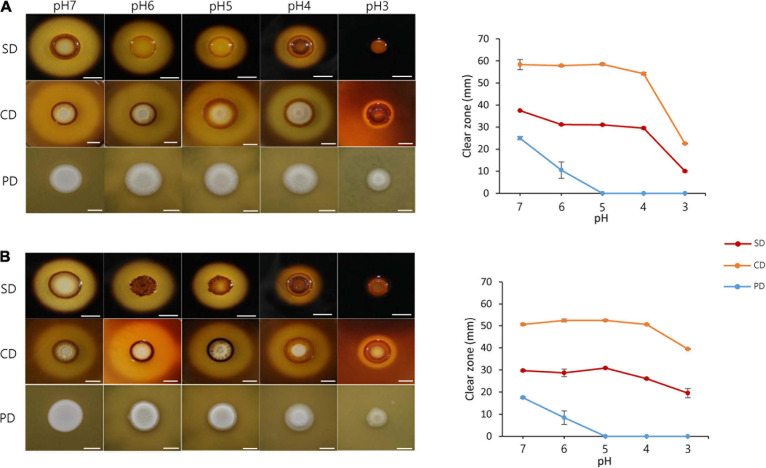
Starch, cellulose, and protein degradation by *Saccharomycopsis fibuligera*. KPH12 strain **(A)** and KJJ81 interspecies hybrid strain **(B)** grown on YPG agar buffered at pH 7, pH 6, pH 5, pH 4, and pH 3 at 25°C. Degradation assays for starch, cellulose, and proteins were performed by adding 0.2% starch, 0.2% carboxymethylcellulose, and 2% skim milk. The results for starch degradation were obtained 3 days after incubation, and the results for cellulose and protein degradation were obtained 5 days after incubation. SD, starch degradation; CD, cellulose degradation; PD, protein degradation; scale bar = 10 mm.

The effect of pH change on fermentation was examined by investigating the degradation of carbohydrates (e.g., starch and cellulose) and proteins by *S. fibuligera*. Both strains of *S. fibuligera* were grown on plates with different pH values ranging from 7 to 3. The pH of the plate was maintained using specific buffers. *S. fibuligera* showed a significant difference in the ability to degrade starch and carbohydrates depending on pH. The KPH12 strain showed the highest activity for hydrolysis of starch and cellulose at pH 7. The hydrolytic activity gradually decreased with decreasing pH and reached its lowest level at pH 3 (Figure 3A). Similarly, the hybrid strain KJJ81 also showed a linear decline in carbohydrate degradation (Figure 3B). Next, we examined the effect of acidic pH values on protein degradation by both strains and found that protein degradation was low at acidic pH values for both strains (Figures 3A,B), indicating that acidic pH conditions suppress the ability of *S. fibuligera* to degrade proteins.

**FIGURE 4:**
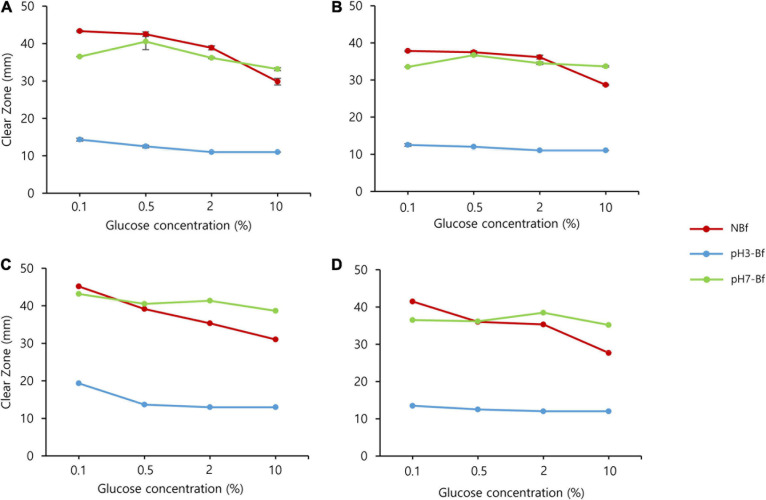
*Saccharomycopsis fibuligera* shows reduced starch and cellulose degradation as pH decreases. Starch **(A,C)** and cellulose degradation **(B,D)** by *Saccharomycopsis fibuligera.* KPH12 **(A,B)** strain and KJJ81 interspecies hybrid strain **(C,D)** were grown on YPG agar plates supplemented with 0.1, 0.5, 2, and 10% glucose in non-buffered (NBf), pH 3-buffered (pH 3-Bf), and pH 7-buffered (pH 7-Bf) conditions. *Red*, non-buffered; *blue*, pH 3-buffered; and *green*, pH 7-buffered.

Next, we examined the effect of glucose concentration on carbohydrate hydrolyzing activity. Both the KPH12 and KJJ81 strains were grown on YPG medium plates supplemented with different glucose concentrations. First, we examined the production of carbohydrate hydrolyzing enzymes. pH had a higher influence on the production of carbohydrate hydrolyzing enzymes than glucose concentration. Starch and cellulose were hydrolyzed at high levels in non-buffered medium supplemented with a low glucose concentration. However, hydrolysis was remarkably reduced in both strains when the glucose concentration was increased, particularly to 10% glucose (Figure 4 and Supplementary Figure 2).

Next, we examined the effect of pH on the hydrolysis activity. We examined the hydrolysis activity at pH 3 or pH 7 and found that the hydrolysis activity of starch and cellulose was inhibited at pH 3 and stimulated at pH 7, regardless of the glucose concentration (Figure 4 and Supplementary Figure 2), thus indicating that pH has a stronger influence than glucose in determining the levels of hydrolyzing activity.

### pH Change Greatly Affects Gene Expression in *S. fibuligera*

Based on previous results, we showed that the morphological and enzymatic alterations occurring during fermentation in *S. fibuligera* are caused by the change in pH. To investigate the effect of the changes in pH that occur during fermentation on the physiology of *S. fibuligera*, we analyzed the transcriptome of *S. fibuligera* at 5 different pH values (pH 3 to pH 7). Total RNA samples were prepared from the cultures, and poly (A)-enriched RNA samples were subjected to high-throughput Illumina HiSeq 2500 sequencing. We obtained 18–40 million reads with Q30 percentages ranging from 88 to over 92%. After alignment with reference genomes of KPH12 and KJJ8, the transcripts were annotated, and out of 6,231 and 12,341 genes annotated in the *S. fibuligera* KPH12 and KJJ81 strains, 6,075 (Supplementary Table 1) and 12,026 (Supplementary Table 2) were expressed, respectively.

**FIGURE 5:**
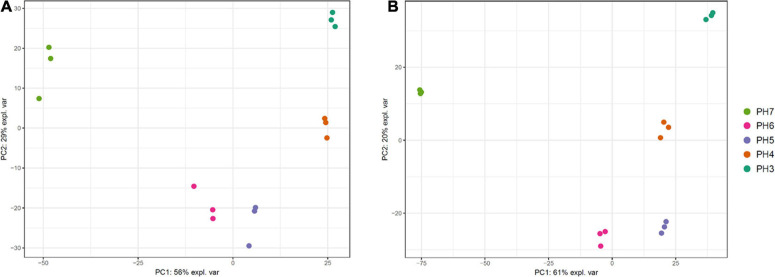
Multidimensional scaling (MDS) plot of 15 RNA samples of *Saccharomycopsis fibuligera*. KPH12 strain **(A)** and KJJ81 interspecies hybrid strain **(B)** were grown at five different pH values for 18 h after inoculation, and total mRNA was prepared and sequenced. *Dot colors*: each dot color indicates the samples at the same pH point.

Multidimensional scaling (MDS) analysis was performed with the RNA samples of each strain to estimate the variability among the samples. We found that the samples aggregated on the PCs based on the pH point. The three replicates at each pH point were clustered together (Figure 5). PC1 accounted for 56–61% of the total variation among the samples, and PC2 accounted for limited variations (20–29%). This result indicates high variation between different pH conditions as well as relatively low variation among replicates.

### Detection of Differentially Expressed Genes (DEGs)

**FIGURE 6:**
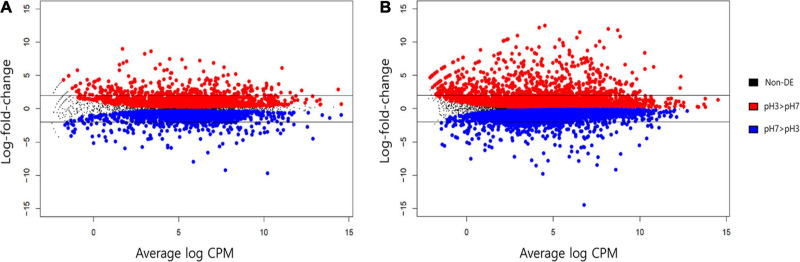
MD plot analysis for the average expression level of all genes of *Saccharomycopsis fibuligera*. KPH12 strain **(A)** and KJJ81 interspecies hybrid strain **(B)** at pH 3 and the log_2_ fold-change of each gene from pH 3 to pH 7. *Blue*: genes with a significant decrease in expression at an FDR of 5%; and *red*: genes with a significant increase in expression at an FDR of 5%. Non-significant genes are in black. Horizontal black bars indicate genes that exhibited at least a fourfold change in expression (log_2_FC = 2).

**FIGURE 7:**
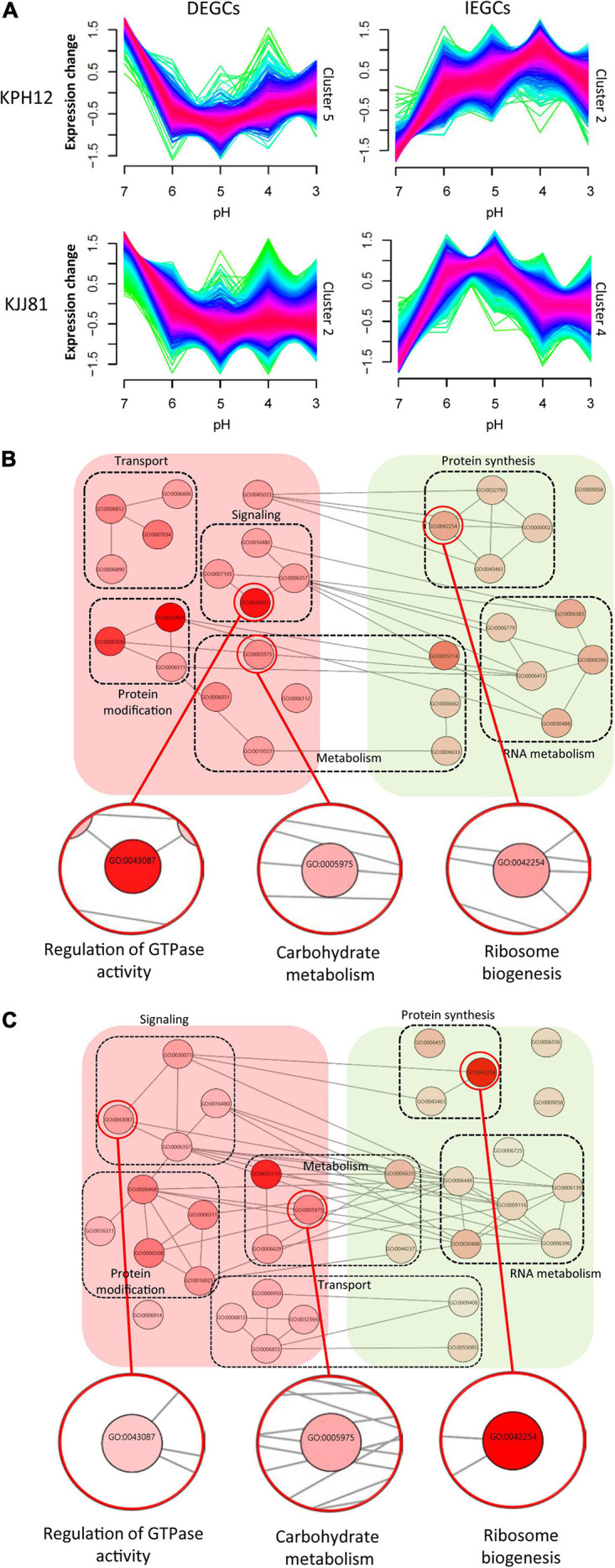
Fuzzy c-means clustering identifies general gene expression patterns of *Saccharomycopsis fibuligera*. **(A)** Clusters of DEGCs and IEGCs of KPH12 and KJJ81 strains at pH values from 7 to 3. Among the five gene clusters classified by fuzzy c-means, two clusters that showed extreme upregulation and downregulation at pH 6 were selected. The membership of a given gene within a cluster is represented by color, with red indicating high association. **(B,C)** For each cluster, enriched biological processes were identified, and only those belonging to the chosen clusters were visualized based on REVIGO. Nodes represent the terms, and edges represent the strength of the terms pairwise similarity. Significance is represented by node color. DEGCs, decreasingly expressed gene clusters; IEGCs, increasingly expressed gene clusters.

At the beginning and end of fermentation, the pH values were 7 and 3, respectively. At these two pH values, *S. fibuligera* showed the clearest difference in hydrolytic enzyme production and morphology. Thus, we determined the genes differentially expressed at the two pH values (pH 7 and pH 3). The EdgeR package in R was used for differentially expressed gene (DEG) determination based on a FDR less than 0.05. Accordingly, 3,867 out of 6,075 genes (Figure 6A and Supplementary Table 3) and 9,176 out of 12,026 genes (Figure 6B and Supplementary Table 4) were detected as DEGs in the transcriptomes of the KPH12 and KJJ81 strains, respectively. To identify the DEGs that were extremely up- and downregulated between pH 7 and pH 6, we employed fuzzy c-means clustering for all DEGs of each strain and investigated the enrichment of the biological processes (BPs) of each cluster. We grouped 3,867 DEGs and 9,176 DEGs of KPH12 and KJJ81 into five clusters (Supplementary Figure 3), respectively. For the KPH12 (Supplementary Table 5) and KJJ81 (Supplementary Table 6) strains, each cluster displayed a unique expression pattern and had significant BPs. Among them, we selected the clustered genes whose expression was highly increased and decreased between pH 7 and pH 6; cluster 2 and cluster 5 of KPH12 and cluster 4 and cluster 2 of KJJ81 contained highly upregulated and highly downregulated genes, respectively (Figure 7A).

### Expression Pattern and Biological Processes of the Selected Clusters

The expression patterns of cluster 5 of KPH12 and cluster 2 of KJJ81 were similar to each other and represented the DEGCs (Figure 7A). The expression of the genes in these clusters was highest at pH 7 and then rapidly declined at pH 6 by more than twofold. The BPs enriched in these clusters were investigated using the R package topGO, filtered and visualized using REVIGO (Supek et al., 2011). The BPs related to transporting cellular components, signaling, protein modification, and metabolism were predominant in the DEGCs of both strains (Figures 7B,C and Supplementary Figure 4A). The BP of carbohydrate metabolism was also found in these clusters, indicating that both strains share many common features in gene regulation and that carbohydrate metabolism is highly influenced by pH. In addition, among the BPs identified by the clusters, a few differences were found between the two strains. For example, the regulation of GTPase activity was higher in KPH12 than in KJJ81.

Unlike DEGCs, the expression patterns of clusters 2 and 4, which represented the increasingly expressed gene clusters (IEGCs) of KPH12 and KJJ81, respectively, were different from each other. The expression of the genes in cluster 2 of the KPH12 strain was extremely low at pH 7 and then increased rapidly to reach the highest level at pH 4, whereas the expression of the genes in cluster 4 of KJJ81 was at the highest level at pH 4 (Figure 7A). BPs that were predominant in these clusters of both strains were related to protein synthesis and RNA metabolism. The gene number of ribosome biogenesis-related BP was higher in KJJ81 than KPH12 (Figures 7B,C, Supplementary Figure 4A, and Supplementary Tables 5, 6). These results suggest that adaptation to acidic pH involves a change in metabolism.

The KEGG pathway enrichment analysis revealed that the DEGs were enriched in arginine biosynthesis, aminoacyl-tRNA biosynthesis, starch and sucrose metabolism in both strains (Supplementary Figure 4B). In strain KJJ81, the upregulated genes were clustered in the fatty acid biosynthesis and N-glycan biosynthesis pathways and the downregulated genes were enriched in starch and sucrose metabolism. In strain KPH12, the upregulated genes were enriched in the lipopolysaccharide biosynthesis pathway and the downregulated genes were clustered in amino acid biosynthesis pathways (Supplementary Figure 4B).

### Expression of the Genes Involved in Carbohydrate Degradation

**FIGURE 8:**
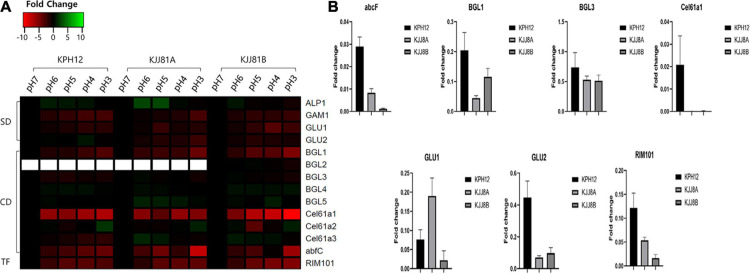
Expression profiles of *Saccharomycopsis fibuligera* genes associated with starch and cellulose degradation as well as the transcription factor RIM101. **(A)** Genes encoding the hydrolytic enzymes were analyzed using the data from RNA-Seq for their expression at the five pH values and presented as a heat map. pH 7 was considered the starting point (log_2_ FC = 0), and the expression change at each pH was calculated based on pH 7. SD, starch degradation; CD, cellulose degradation; TF, transcription factor. **(B)** qRT-PCR analysis. To confirm the results obtained from the RNA-seq data, a qRT-PCR assay was performed for the indicated genes using specific primers. Error bar, standard deviation (*n* = 3).

In *S. fibuligera*, we investigated the expression of genes previously known to be involved in starch degradation, such as *a*-amylase (*ALP1*) and glucoamylases (*GAM1* and *GLU*), and those involved in cellulose degradation, such as β-glucosidases (*BGL*), polysaccharide monooxygenase (*Cel61a*) and alpha-L-arabinofuranosidase C (*abfC*). Most of the genes involved in starch degradation were downregulated (Figure 8A). Three of these genes, *GAM1* (KPH12A7G027400, KJJ81A4G036700, KJJ81B7G026300), *GLU1* (KPH12A1G148700, KJJ81A6G044500, KJJ81B1G146400) and *GLU2* (KPH12A6G044000, KJJ81A6G044500, KJJ81B 6G043300), are among the DEGs of KPH12 and KJJ81. We confirmed the downregulation of most of these genes at pH 3 by qRT-PCR (Figure 8B). All these genes were present in clusters 1 and 5 except *GLU2*, which was present in clusters 2 and 3 in KPH12 (Supplementary Figure 3A). On the other hand, most of these genes were present in cluster 2 of the DEGCs and in cluster 5 in both the A and B genomes of hybrid strain KJJ81 (Figure 7A). Only *GAM1* was located in different clusters, and it was in cluster 5 of genome A and cluster 2 of genome B (Supplementary Figure 3A).

Among the genes related to cellulose degradation, five out of the 8 genes in genome A and 6 out of 9 in genome B were downregulated in the two strains (Figure 8A). All the downregulated genes of KPH12 were located in DEGs. Among them, *BGL3* and *Cel61a1* were in cluster 5 of the DEGCs, whereas the remaining 3 genes, *BGL1* (KPH12A2G002200, KJJ81A2G002300, KJJ81B2G002200), *Cel61a3* (KPH12A2G080500, KJJ81A2G080600, KJJ81B2 G078400), and *abfC* (KPH12A3G089600, KJJ81A3G090700, KJJ81B5G075900), were in cluster 1 (Supplementary Figure 3A). On the other hand, these genes were downregulated in the hybrid strains and were present in the DEGs except *Cel61a3* of genome B. Two of these DEGs, *BGL1* and *Cel61a1*, were found in cluster 2, and *BGL3* was in cluster 5 (Supplementary Figure 3A). *abfC* of genome A was found in cluster 2 of the DEGCs (Figure 7A), while that of genome B was not found in any cluster. The reduced expression of most of these genes was confirmed by qRT-PCR (Figure 8B). *RIM101* (KPH12A2G072500, KJJ81A2G072500, KJJ81B2G070400), a pH-response transcription factor, was associated with the expression of starch and cellulose degradation-related genes (Figure 8A). Thus, we examined its expression in both strains under different pH conditions. *RIM101* was found in clusters 5 and 2 of the DEGCs in KPH12 and KJJ81, respectively. We confirmed the low expression of *RIM101* at pH 3 by qRT-PCR, which was consistent with the RNA-Seq data (Figure 8B).

### Expression of the Genes Involved in the Dimorphism

**FIGURE 9:**
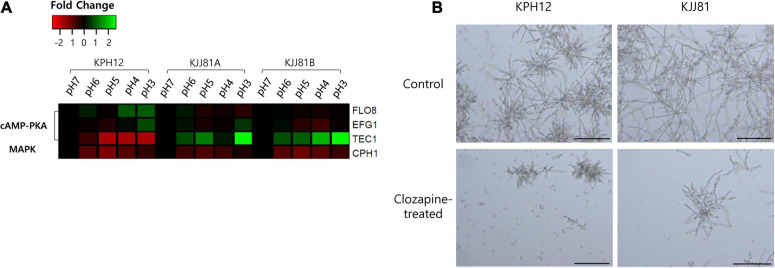
Expression profiles of *Saccharomycopsis fibuligera* transcriptional activators of cAMP-PKA and MAPK, the two signaling pathways associated with the dimorphic growth of several model yeasts and fungi, at each pH point. pH 7 was considered the starting point (log_2_ FC = 0), and the expression at each pH was calculated based on pH 7. **(A)** shows the RNA-Seq data and **(B)** shows the cell morphology of *S. fibuligera* strains in the presence or absence of the cAMP-PKA blocker clozapine. SD, starch degradation; CD, cellulose degradation; TF, transcription factor; scale bar = 100 μm.

cAMP-PKA and MAPK cascades are well-known signaling pathways involved in the induction of pseudohyphal growth of the model yeast *S. cerevisiae* (Pan et al., 2000) and the hyphal or yeast form in other dimorphic fungal models (Leberer et al., 2001; Martínez-Espinoza et al., 2004). We examined the expression of homologous genes in *S. fibuligera* that are involved in these cascades, such as transcriptional activator *FLO8* (KPH12A6G0101800, KJJ81A6G018300), enhanced filamentous growth *EFG1* (KPH12A1G008700, KJJ81A1G009000, KJJ81B1G008600), and transcription factor *TEC1* (KPH12A7G016700, KJJ81A7G016700, KJJ81B7G015800), for cAMP-PKA and transcription factor *CPH1* (KPH12A3G005900, KJJ81A3G005800, KJJ81B3G006100) for MAPK. These transcriptional activators were among the DEGs except for *FLO8* and *TEC1*. The expression of *CPH1* was slightly downregulated in KPH12 and the two genomes of the hybrid strain KJJ81 (Figure 9A), and at least one of the cAMP-PKA transcriptional activators *FLO8*, *EFG1*, or *TEC1* was upregulated (Figure 9A). These results suggest that the cAMP-PKA pathway is involved in the hyphal growth of *S. fibuligera*. To confirm this supposition, KPH12 and KJJ81 cells were grown in YPG broth media buffered at pH 4 in the presence or absence of clozapine, which blocks the cAMP-PKA signaling pathway (Midkiff et al., 2011), and the morphology was examined. In the liquid cultures, hyphal growth was suppressed in both strains by up to 60.4 and 26.2% for KPH12 and KJJ81, respectively, in the presence of clozapine (Figure 9B), indicating that the cAMP-PKA pathway plays a pivotal role in hyphal induction in both strains, KPH12 and KJJ81.

## Discussion

In this study, we investigated the growth of *S. fibuligera* and production of hydrolytic enzymes during fermentation. During *makgeolli* production, the microorganisms originating from *nuruk* affect the environmental conditions of fermentation and vice versa. We focused on *S. fibuligera* because this microorganism is one of the most predominant amylolytic and cellulose-degrading yeasts in *nuruk* (Carroll et al., 2017). We found that *S. fibuligera* undergoes several phenotypic changes, such as the dimorphic transition as the pH goes down. Additionally, we showed that acidic conditions (pH 5–3) induce the yeast-to-hyphal transition of *S. fibuligera*, whereas neutral conditions (pH 7) induce yeast growth. Thus, the morphological change of *S. fibuligera* is consistent with the pH decline that is observed on the second day of *makgeolli* fermentation (Song et al., 2015). The morphological change of *S. fibuligera* was similar to that of *U*. *maydis*, which grows as mycelium at pH 3 and as yeast at pH 7 (Ruiz-Herrera et al., 1995), but differs from that of *Y. lipolytica* and *C. ablicans*, which grow as yeast at acidic pH and as hyphae under neutral pH conditions (Ruiz-Herrera and Sentandreu, 2002; Davis, 2003). Compared with the parental strains, we observed that the hybrid strain underwent hyperfilamentation, which might be another kind of adaptation to acidic pH. However, Choo and coworkers (Choo et al., 2016) found that a high concentration of glucose also induced the hyphal growth of the two strains of *S. fibuligera*, KPH12 and KJJ81.

The yeast to hyphal transition of *S. fibuligera* during fermentation was further examined at the level of gene expression. The expression patterns of genes involved in the dimorphic transition in *S. fibuligera* were investigated through transcriptome analysis. The Rim101/Pac signaling pathway plays an essential role during the dimorphic transition in response to alkaline pH (Davis, 2009). As shown in Figure 8, the *RIM101* gene was upregulated at neutral pH and downregulated at acidic pH. Wang et al. (2020) found that in *Trichosporon cutaneum*, neutral and acidic pH induces and inhibit the expression of *RIM101*, respectively, which correlates with our findings. Under alkaline conditions, *C. albicans* senses pH signals through the membrane receptor *RIM21*, which activates the arresting protein *RIM8*. *RIM8* interacts with the endosomal sorting complexes required for transport (ESCRT) complex and stimulates the expression of *RIM20* (zinc finger transcription factor) and *RIM13* (calpain protease) (Henne et al., 2011). *RIM20* and RIM13 form a complex that hydrolyzes the long form of RIM101 into a short form (Garnaud et al., 2018). The short form of *RIM101* induces the expression of pH-responsive protein 1 (*PHR1*), which plays an important role in growth at alkaline pH (Saporito-Irwin et al., 1995), and suppresses the expression of *PHR2* (pH-responsive protein 2) (Baek et al., 2006). PHR2 is induced at acidic pH and required for infection by *C. albicans*. In our study, the meiotic activator *RIM4* (KPH12A1G023400) and pH-response regulator protein *RIM20* (KPH12A4G038700) were downregulated under acidic pH conditions along with the key regulator *RIM101* (KPH12A2G072500) in *S. fibuligera* KPH12 (Supplementary Table 8). In addition, the expression patterns of *RIM101* (KJJ81A2G072500), *RIM4* (KJJ81A1G023900) and *RIM20* (KJJ81B4G038300) in the hybrid strain KJJ81 were similar to those in *S. fibuligera* KPH12. These results may indicate conservation of the RIM101 pathway in *S. fibuligera*. Furthermore, the transcriptional repressor of filamentous growth *TUP1* (KPH12A4G054300), which is under the control of the RIM101 pathway, was downregulated at acidic pH (Supplementary Table 8). *TUP1* forms with the general transcriptional corepressor *SNN6* a complex repressor (TUP1-SSN6), which is conserved in eukaryotes. The TUP1-SSN6 complex plays a key role in growth regulation in several dimorphic fungi (Smith and Johnson, 2000) by modulating sequence-specific DNA-binding proteins (DBPs). Among the DPBs, *MIG1* (regulator protein MIG1 involved in glucose repression), *NRG1* (transcriptional repressor involved in regulation of glucose repression) and *RFG1* (repressor of filamentous growth 1) inhibit filament-inducing genes by binding to the TUP1-SSN6 complex in *C. albicans* (Kebaara et al., 2008; Sánchez-Arreguin et al., 2021). Compared with the results of previous studies, the current study found that *NRG1* (KPH12A4G012800, KPH12A2G036800, KJJ81A3G036900, KJJ81B3G035800) and *RFG1* (KPH12A2G100300, KJJ81B2G097900) were upregulated in *S. fibuligera* under acidic pH conditions (Supplementary Tables 8, 9). These results suggest that *NRG1* and *RFG1* could play different roles during changes in environmental pH.

In addition to transcription factors, we found that some DEGs involved in the cAMP/PKA pathway were differentially expressed (Figure 9 and Supplementary Figure 4). Cyclic AMP-protein kinase (cAMP/PKA) and MAPK are two signaling pathways that play a central role in the regulation of morphogenesis in several fungi, including *S. cerevisiae* (Conrad et al., 2014), *C. albicans* (Lin and Chen, 2018), and *T. cutaneum* (Wang et al., 2020). However, the two signaling pathways are not functionally conserved among various fungi. For instance, both signaling pathways are involved in the induction of the pseudohyphal and hyphal growth of *S. cerevisiae* and *C. albicans*, respectively (Sudbery, 2011), whereas in *Y. lipolytica*, only MAPK is involved in the induction of hyphal growth while cAMP/PKA induces yeast growth (Ruiz-Herrera and Sentandreu, 2002; Cervantes-Chávez and Ruiz-Herrera, 2006; Martínez-Soto and Ruiz-Herrera, 2015). In *U. maydis*, hyphal growth is induced only by cAMP/PKA and yeast growth is induced by MAPK (Martínez-Espinoza et al., 2004). Alkaline pH signals induce the activation of adenylate cyclase (*CYR1*) and *PKA* through *RAS2* and *GPA2*, which leads to the activation of transcription factors, such *RGT1*, *EFG1* and *TEC1*, in *C. albicans* (Zhang et al., 2016). These transcription factors play a key role in hyphal growth, and their activation leads to translation from yeast to hyphal form (Han et al., 2011; Cornet and Gaillardin, 2014). In this study, the expression of G-protein alpha-subunit (*GPA2*, KJJ81B5G067700), Ras-like protein 1 (*RAS1*, KJJ81B2G095300), and G protein-coupled receptor 1 (*GPR1*, KJJ81B4G062500) involved in the cAMP/PKA pathway was upregulated at acidic pH in hybrid strain KJJ81 (Supplementary Table 9). Furthermore, the transcription factors *TEC1* (KJJ81A7G016700, KJJ81B7G015800) and *EFG1* (KPH12A1G008700) were upregulated under acidic conditions (Supplementary Tables 8, 9), which is consistent with previous studies in *T. cutaneum* (Wang et al., 2020). Thus, these results suggest that the cAMP/PKA pathway is involved in the regulation of hyphal growth at acidic pH in the hybrid strain *S. fibuligera* KJJ81 (Figure 9 and Supplementary Table 9).

During fermentation, the most crucial process is the degradation of carbohydrates in *nuruk* to produce substrates of alcohol. Thus, the production of hydrolyzing enzymes should be crucial for successful fermentation. However, as fermentation progresses, the pH drops, which in turn leads to a decrease in the production of hydrolytic enzymes for starch, cellulose and proteins in *S. fibuligera* (Figure 3), and these conditions are not favorable for fermentation. In addition, a high concentration of glucose suppresses the production of hydrolytic enzymes for starch, protein, and cellulose in *S. fibuligera* (Chi et al., 2009). Choo et al. (2016) showed that the reduced production of these enzymes under high concentrations of glucose is caused by the downregulation of the genes encoding hydrolytic enzymes. A previous study showed that a high concentration of glucose changed the environmental pH to alkaline conditions in *C. albicans* (Carman, 2008). Thus, it is not clear whether a high glucose concentration directly affects the production of hydrolytic enzymes. However, in this work, we showed that the pH change resulting from glucose utilization but not glucose itself plays a critical role in the dimorphic transition and the reduced production of hydrolytic enzymes in *S. fibuligera* (Supplementary Figure 2). The transcriptome analysis revealed that genes encoding enzymes involved in starch, cellulose and protein degradation were downregulated at acidic pH (Figure 8). *ALP1* (KPH12A6G028500, KJJ81A6G028500, KJJ81B6G028100), *GAM1* (KPH12A7G02 7400, KJJ81A7G027600, KJJ81B7G026300), *GLU1* (KPH1 2A1G148700, KJJ81A1G148800, KJJ81B1G146400) and *GLU2* (KPH12A6G044000, KJJ81A6G044500, KJJ81B6G043300), which encode enzymes involved in starch degradation, were downregulated at acidic pH compared to alkaline pH (Supplementary Tables 8, 9). In addition, the expression of *RIM101* was decreased at acidic pH. In the model filamentous organism *Aspergillus nidulans*, the deletion of *RIM101* resulted in a lower expression of carbohydrate degradation-related genes (Peñalva et al., 2008). Thus, it is possible that *RIM101* could be involved in the regulation of carbohydrate degradation-related genes in *S. fibuligera*.

We investigated the expression of genes involved in protein synthesis and RNA metabolism. Through an analysis of BPs, the genes linked to RNA metabolism and protein synthesis, such as *CTK2* (CTD kinase subunit beta, KPH12A5G020700, KJJ81B5G020000), *DBP3* (ATP-dependent RNA helicase DBP3, KPH12A7G005900, KJJ81A7G005800, KJJ81B7G005500) and *CBP6* (KPH12A2G119900, KJJ81A2G119600, KJJ81B2G116300), were upregulated under hyphal induction conditions (Supplementary Tables 8, 9). A transcriptome analysis of *C. albicans* under the condition of transition from yeast to hyphae showed an upregulation of genes involved in RNA metabolism and protein synthesis (Carlisle and Kadosh, 2013). Similar results were observed with *U. maydis* during the transition from yeast to hyphal forms (Larraya et al., 2005). However, contrasting cases are observed as well, and genes linked with protein synthesis and RNA metabolism were involved in the transition to the yeast form in *Paracoccidioides brasiliensis* (Nunes et al., 2005; Parente et al., 2008), *Penicillium marneffei* (a human pathogen) (Yang et al., 2014) and *Ophiostoma novo-ulmi* (a plant pathogen) (Nigg and Bernier, 2016). *C. albicans* and *U. maydis* exist in yeast form as saprotrophs in the environment (Vollmeister et al., 2011; Carlisle and Kadosh, 2013; Ruiz-Herrera et al., 2020), and they turn into filamentous forms to infect their hosts. In contrast, the opposite is true for *P. brasiliensis*, *P. marneffei* and *O. novo-ulmi* (Rooney and Klein, 2002; Naruzawa and Bernier, 2014). Therefore, the high-level expression of genes involved in protein synthesis and RNA metabolism may not be a direct indicator of the transition from yeast to hyphae but rather an indication of adaptation to changing environmental conditions. Accordingly, the transition of *S. fibuligera* from yeast to hyphae is an adaptation to acidic pH supported by the high-level expression of genes related to protein synthesis and RNA metabolism. In addition, cell wall synthesis is tightly regulated under hyphal formation conditions. *BCR1* (biofilm and cell wall regulator 1) is a transcription factor that plays a key role in the regulation of cell wall formation and regulates the expression of genes involved in cell wall synthesis (*HWP1*, *ALS1*, *ALS3*, and *HYR1*) (Nobile et al., 2006; Sosinska et al., 2011). The deletion of *BCR1* in *C. albicans* induced a decrease in the expression of the cell wall chitinase *Cht2* (Sherrington et al., 2017). Under acidic conditions, the *S. fibuligera* hybrid strain KJJ81 showed an upregulation of *BCR1* (Supplementary Table 9, KJJ81B4G072900), indicating that the function of *BCR1* is conserved and may play an important role in yeast-hyphal transition through regulation of cell wall synthesis and regulation of the wall proteome during adaptation.

## Conclusion

In conclusion, the study provides evidence that the acidic pH conditions that occur during fermentation of *nuruk* underlie the phenotypes of *S. fibuligera*, such as the lower-level starch and cellulose degradation and hyphal growth. Here, we also provide the first detailed analysis of transcriptome changes in *S. fibuligera* upon pH decline. During the first phase of fermentation of *nuruk*, *S. fibuligera* contributes to the degradation of starch through secretion of carbohydrate-hydrolyzing enzymes. As fermentation progresses, the environmental pH drops to an acidic level (pH 3). Under these conditions, *S. fibuligera* undergoes a morphological transition to give rise to the hyphal form and metabolic modifications, such as inhibition of carbohydrate metabolism, to survive under acidic pH conditions as a means of adapting to fermentation environments. In addition, our transcriptome analysis contributes to elucidating some aspects of the dimorphism transition of *S. fibuligera* under low pH conditions that occur when fermenting *makgeolli* using *nuruk*.

## Data Availability Statement

The datasets presented in this study can be found in online repositories. The names of the repository/repositories and accession number(s) can be found below: https://www.ncbi.nlm.nih.gov/, PRJNA705233.

## Author Contributions

J-AS conceived the conceptualization, funding acquisition, project administration, supervision, writing the original draft, responding to revision, and editing the whole manuscript. MF performed the experiment and the RNA-sequence analysis, and drafted the first manuscript. NA participated in the RNA-seq analysis and drafted the manuscript. All authors contributed to the article and approved the submitted version.

## Conflict of Interest

The authors declare that the research was conducted in the absence of any commercial or financial relationships that could be construed as a potential conflict of interest.
