# Supplementary material for: pH Changes Have a Profound Effect on Gene Expression, Hydrolytic Enzyme Production, and Dimorphism in Saccharomycopsis fibuligera
Source: Front Microbiol. 2021 Jun 24;12:672661. doi: 10.3389/fmicb.2021.672661 (PMC8265565; doi:10.3389/fmicb.2021.672661)
Supplement: Supplementary file 2 [file Data_Sheet_1.docx]

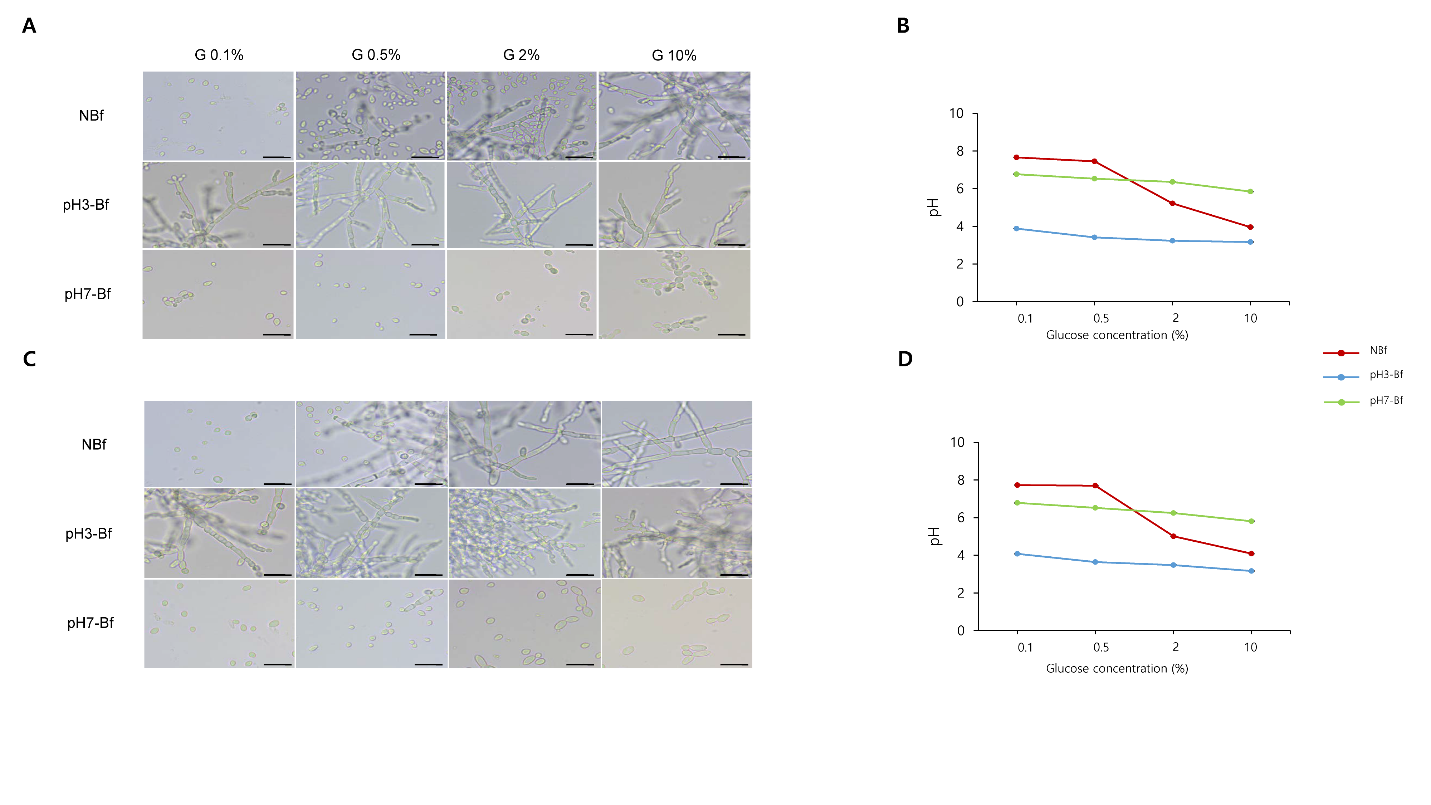


**Supplementary Figure 1.** Cell morphology and the pH of the medium of KPH12 strain and KJJ81 interspecies hybrid strain. Cells of KPH12 strain (**A, B**) and KJJ81 (**C, D**) were grown in YP broth supplemented with 0.1%, 0.5%, 2%, and 10% glucose in non-buffered (NBf), pH 3-buffered (pH 3-Bf), and pH 7-buffered (pH 7-Bf) conditions. The morphology (**A, C**) and growth medium pH (**B, D**) were examined. Red: non-buffered; blue: pH 3-buffered; green: pH 7-buffered; G: glucose; Bars are equal for 20 μm


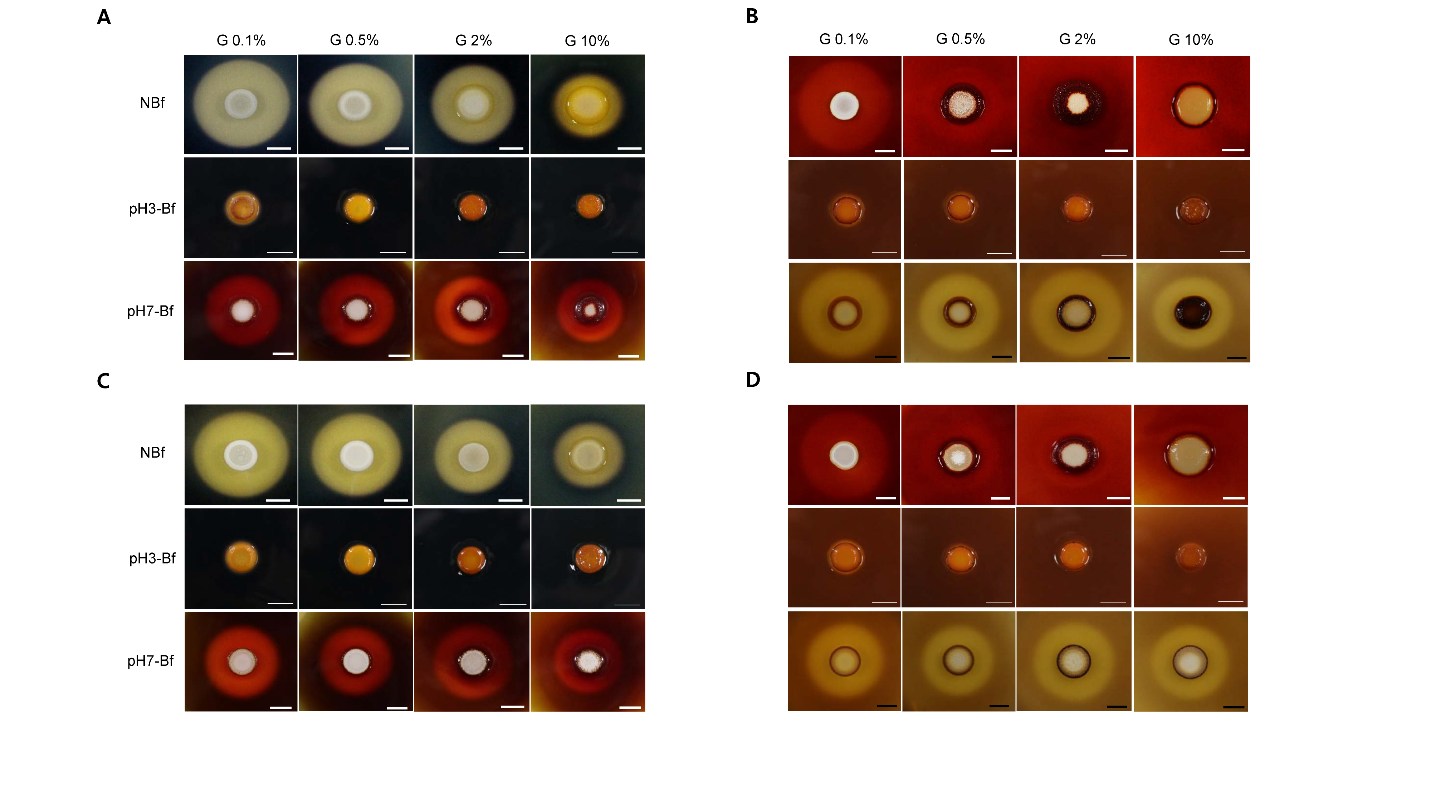


**Supplementary Figure 2.** Starch and cellulose degradation by *Saccharomycopsis fibuligera*. KPH12 strain (**A, B**) and KJJ81 interspecies hybrid strain (**C, D)** grown on in YP agar supplied with 0.1%, 0.5%, 2%, and 10% glucose in non-buffered (NBf), pH 3-buffered (pH 3-Bf), and pH 7-buffered (pH 7-Bf) conditions. G: glucose; Scale bars = 10 mm.


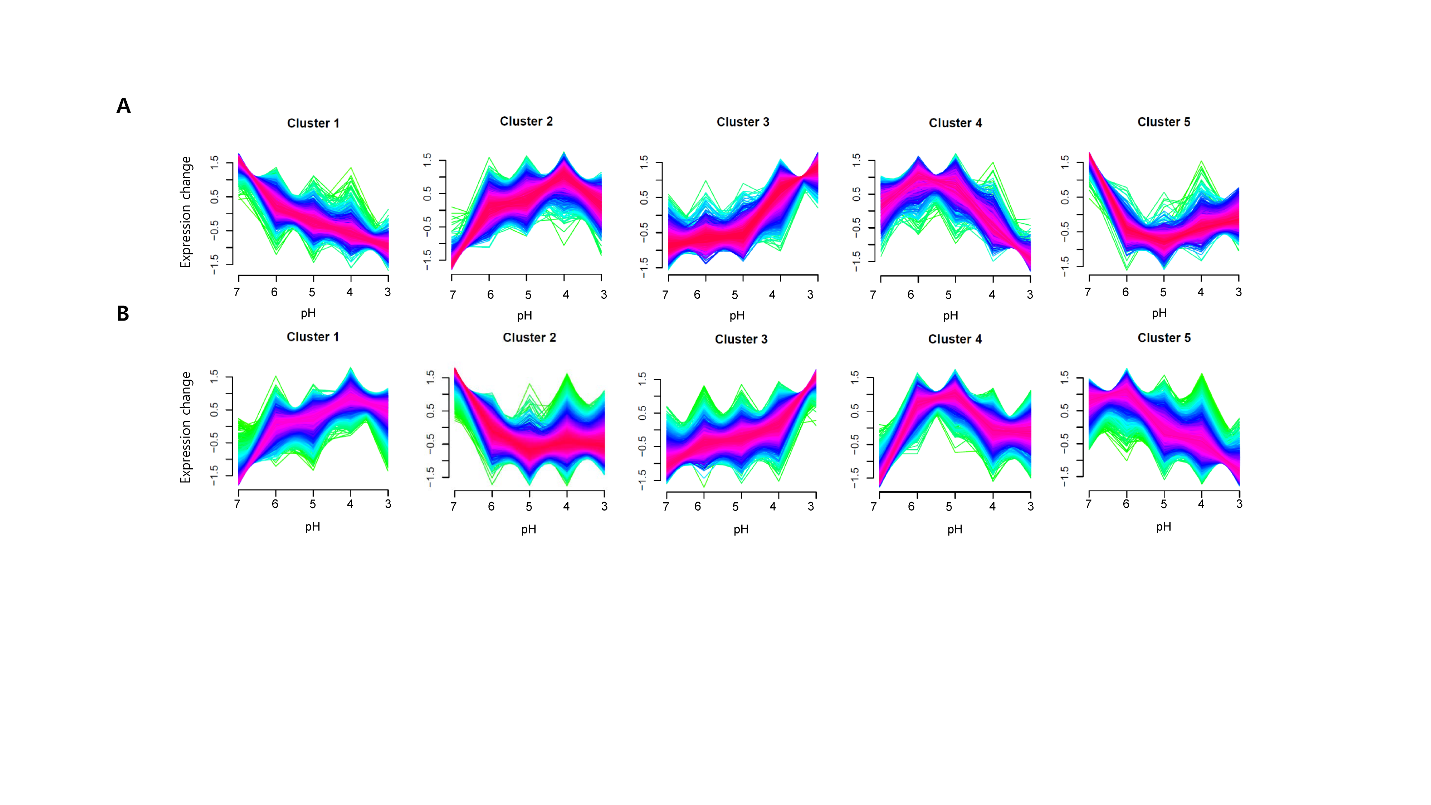


**Supplementary Figure 3.** Fuzzy c-means clustering analysis for the effect of pH values on gene expression in *S. fibuligera* KPH12 and KJJ81 strains. Fuzzy c-means clustering analysis was carried out to identify general patterns of gene expression of *S. fibuligera* KPH12 and KJJ81 strains across the pH development from pH 7 to pH3.


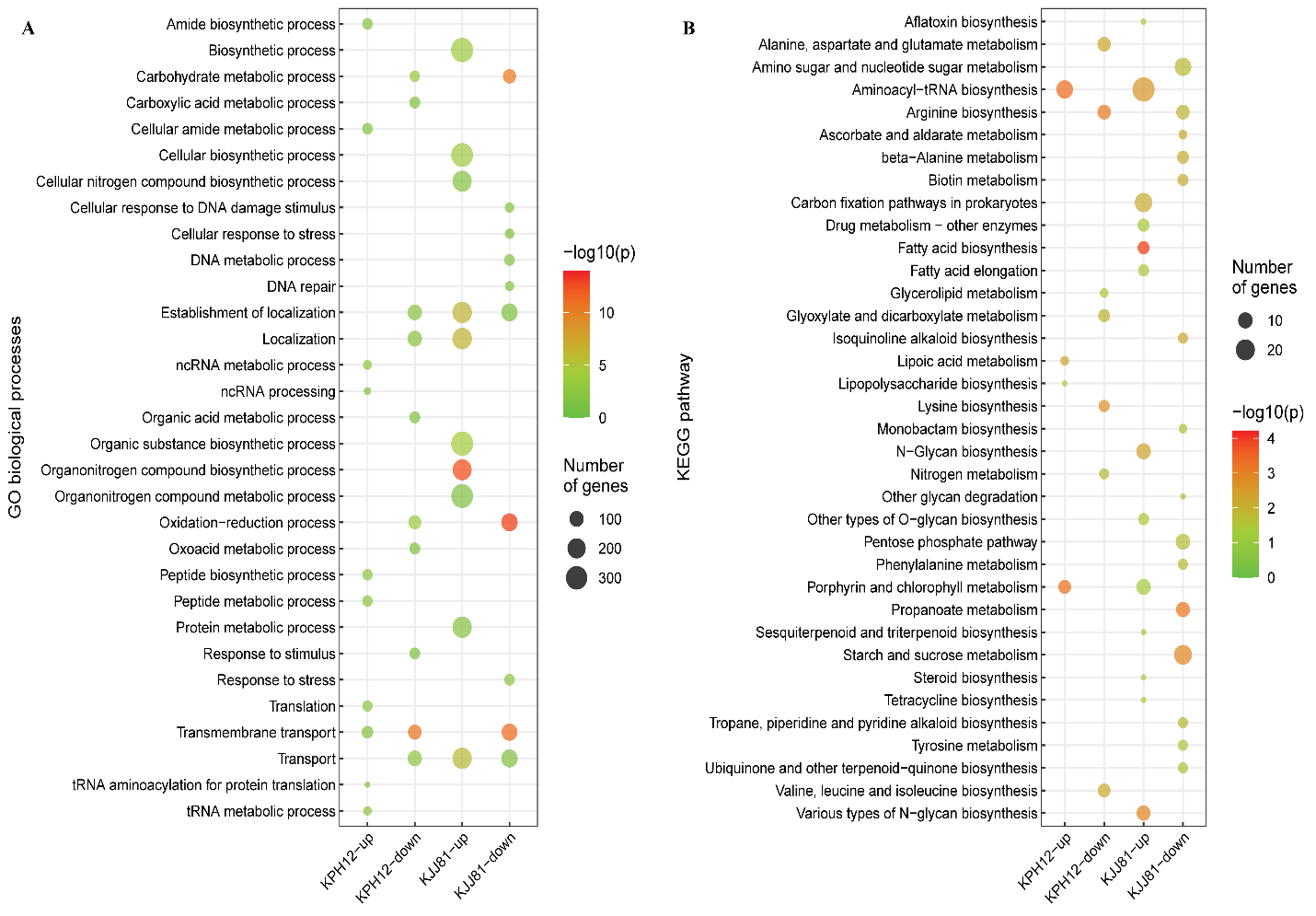


**Supplementary Figure 4. (A)** Top Gene Ontology terms of differentially expressed genes. **(B)** KEGG pathway enrichment analysis of differentially expressed genes. Y-axis label represent GO term or KEGG pathway, and X-axis label indicates the downregulated and upregulated genes for *S. fibuligera* KPH12 and KJJ81 strains. The dot size indicates the number of genes differentially expressed enriched in the GO or the KEGG pathway and color indicates the enrichment significance.

**Tables S1-S9. Supplementary Table 1:** Normalized counts of the all expressed genes of *Saccharomycopsis fibuligera* KPH12 strain. **Supplementary Table 2:** Normalized counts of the all expressed genes of *Saccharomycopsis fibuligera* KJJ81 strain. **Supplementary Table 3:** Normalized counts of the 3867 DEG of *Saccharomycopsis fibuligera* KPH12 strain determined using EdgeR, FDR < 0.05. **Supplementary Table 4:** Normalized counts of the 9176 DEG of *Saccharomycopsis fibuligera* KJJ81 strain determined using EdgeR, FDR < 0.05. **Supplementary Table 5:** Enriched biological processes of the five clusters of *Saccharomycopsis fibuligera* KPH12 strain's DEG using TopGO and REVIGO. GO term. **Supplementary Table 6:** Enriched biological processes of the five clusters of *Saccharomycopsis fibuligera* KJJ81 strain's DEG using TopGO and REVIGO. GO term. **Supplementary Table 7:** Information of qRT-PCR primers used in this study. **Supplementary Table 8-9**: Fold change of unigenes at different pH values and their functional annotation in *S. fibuligera* KPH12 and KJJ81. **Supplementary Table 10**: Percentage of yeast cells in each *Saccharomycopsis fibuligera* KPH12 and KJJ81 grown on Non-buffered, pH3-buffered and pH7-buffered YPG with different concentrations of glucose (0.1, 0.5, 2, and 10%). **Supplementary Table 11-12**: Functional annotation of predicted genes and their respective position at genome level in the *Saccharomycopsis fibuligera* KPH12 strain and KJJ81 strain with their nucleotide and protein sequences.
